# Control of Communicable Diseases in Human and in Animal Populations: 70th Anniversary of the Year of the Birth of Professor Rick Speare (2 August 1947–5 June 2016)

**DOI:** 10.3390/tropicalmed3040106

**Published:** 2018-09-28

**Authors:** Jorg Heukelbach

**Affiliations:** 1 Department of Community Health, School of Medicine, Federal University of Ceará, Fortaleza CE 60430-140, Brazil; heukelbach@web.de; 2 College of Public Health, Medical and Veterinary Sciences, Division of Tropical Health and Medicine, James Cook University, Townsville 4811, Australia

This special issue is dedicated to the memory of Prof. Rick Speare, whose academic contribution included high level research of human and veterinarian medical interest on zoonotic diseases and public health in general, following the One Health approach. He dedicated much of his work to Aboriginal communities. In 2016, Rick was tragically killed in a car crash while driving to a seminar at James Cook University in Queensland, Australia.

This special issue contains a total of 17 peer-reviewed papers (9 original papers, 1 case report, 7 reviews), many of them published by Rick’s former colleagues and co-researchers. Some papers contain material collected together with Rick, which for the first time is published here.

Several papers are on strongyloidiasis—one of Rick’s main research focuses. Wilson & Fearon [1] describe high prevalence of pediatric strongyloidiasis in Australia. Different aspects of diagnosis of strongyloidiasis in hyperendemic regions in northern Australia are presented by Robertson et al. [2]. Another review paper on strongyloidiasis focuses on the disease during pregnancy [3]. The authors describe the effects of strongyloidiasis, such as low birth weight and anemia, in children and pregnant women. Page et al. [4] discuss public health issues and intervention measures, considering the particular life cycle of *Strongyloides stercoralis*; and Miller et al. [5] present results from a community-based health promotion and control program in an Aboriginal community in central Queensland, Australia, with impressive community involvement. This is the first community-directed strongyloidiasis control program—a work that had been initiated by Rick during his lifetime. Beknazarova et al. [6] discuss the importance of strongyloidiasis being a notifiable disease in Australia, presenting plausible arguments for their case.

Another study from James Cook University investigated immunization rates of medical students [7]. The idea for this study was given by Rick during a lecture. Despite an attitude of high importance of vaccination for personal protection, immunization rates were astonishingly low in the group at in this high risk for contracting communicable diseases.

Gordon et al. [8] review the history of lymphatic filariasis in Australia and Oceania and discuss the possibility of reintroduction of the disease into Australia. In another review, Khan & Zhador [9] give an overview of brucellosis, an important zoonotic disease, and highlight its importance to both human health and livestock. Shima et al. [10] describe two cases of echinococcosis in Lumholtz’s tree-kangaroos. This paper is of particular interest, because Rick Speare was the first person to identify hydatid cysts in a Lumholtz’s tree-kangaroo, although he never published his findings.

Two papers include data on scabies. Ugbomoiko et al. [11] performed a field study in rural communities in Nigeria. Scabies was present in almost two-thirds of the population, and associated morbidity was considerable. They also show that even in resource-poor settings, scabies is associated with extreme poverty. Shield et al. [12] present their work on community education about strongyloidiasis and scabies—two highly endemic diseases in remote aboriginal communities. Using a discovery education approach including cultural knowledge in the local language, they achieved impressive results.

Andrew Thompson’s review on threats to Australia’s biosecurity presents a comprehensive overview on the topic and discusses misconceptions and challenges [13]. A survey on intestinal parasites from dog fecal samples collected in Queensland/Australia detected a variety of parasites, including those with zoonotic potential [14].

Ahmed et al. [15] describe case fatality rates due to diarrhea and respiratory diseases in preschool children in Mauritania. While the rates are shockingly high, this paper fills a knowledge gap from a country where still little evidence is being published. A study from Tanzania assessed the diagnostic performance of an antigen rapid test in diagnosing intestinal schistosomiasis in HIV-1 infected patients, as compared to classical Kato Katz technique [16]. The authors show that in HIV1-infected individuals, Kato Katz technique has a low sensitivity and proposes use of the rapid test in high HIV prevalence settings. A Brazilian paper by de Amorim de Souza et al. [17] describes the epidemiology of leprosy during a period of 14 years. Spatial analyses show that the disease is heterogeneously distributed in a state in Brazil’s northeast region, and the authors argue that control actions should be intensified in focal areas.

Taken together, the diversity of papers, depth of the topics, geographical range, and the inclusion of impressive studies from aboriginal and remote communities published in this special issue perfectly reflect Rick’s enthusiasm, scientific spirit, and diversity. I am very much pleased to share the content with the international scientific community. Rick would have loved to see all these papers being published!
